# Sparse sliced inverse regression for high dimensional data analysis

**DOI:** 10.1186/s12859-022-04700-3

**Published:** 2022-05-07

**Authors:** Haileab Hilafu, Sandra E. Safo

**Affiliations:** 1 Department of Business Analytics and Statistics, University of Tennessee, Knoxville, TN 37996 USA; 2 Division of Biostatistics, University of Minnesota, Minneapolis, MN 55455 USA

**Keywords:** Semiparametric model, Generalized eigenvalue decomposition, Sliced inverse regression, Linear discriminant analysis, High-dimensional data

## Abstract

**Background:**

Dimension reduction and variable selection play a critical role in the analysis of contemporary high-dimensional data. The semi-parametric multi-index model often serves as a reasonable model for analysis of such high-dimensional data. The sliced inverse regression (SIR) method, which can be formulated as a generalized eigenvalue decomposition problem, offers a model-free estimation approach for the indices in the semi-parametric multi-index model. Obtaining sparse estimates of the eigenvectors that constitute the basis matrix that is used to construct the indices is desirable to facilitate variable selection, which in turn facilitates interpretability and model parsimony.

**Results:**

To this end, we propose a group-Dantzig selector type formulation that induces row-sparsity to the sliced inverse regression dimension reduction vectors. Extensive simulation studies are carried out to assess the performance of the proposed method, and compare it with other state of the art methods in the literature.

**Conclusion:**

The proposed method is shown to yield competitive estimation, prediction, and variable selection performance. Three real data applications, including a metabolomics depression study, are presented to demonstrate the method’s effectiveness in practice.

## Background

High-throughput technologies such as microarray and next-generation sequencing have been widely applied in biomedical research to monitor genome-wide DNA, RNA and epigenetic molecular activities and to detect disease-associated events or biomarkers. With decrease in experimental costs over the years, tremendous amounts of data have been generated and accumulated in public data depositories in the past two decades (e.g. Gene Expression Omnibus (GEO) and Sequence Read Archive (SRA) from NCBI, ArrayExpress from EBI, and the NIH Metabolomics Workbench data repository). Perhaps due to limitation of clinical tissue access, individual labs usually generate omics datasets with small to moderate sample sizes (e.g. $n = 40 - 1000$). Statistical power and reproducibility of studies using such large-*p*-small-*n* data has long been a concern. Dimension reduction (feature extraction) and variable selection (feature selection) play a crucial role for down-stream pattern recognition, classification and clustering, with such high-dimensional data. In this article, we propose dimension reduction and variable selection for semi-parametric models in the high-dimensional setting.

Let *Y* denote the response variable and ${{\mathbf{X}}}$ denote the *p*-dimensional predictor vector. Consider the semi-parametric multi-index model

1 $$\begin{aligned} Y = g (\mathbf{v }^{\top }_1{{\mathbf{X}}},\ldots ,\mathbf{v }^{\top }_d{{\mathbf{X}}},\varepsilon ), \end{aligned}$$

where $\mathbf{v }_1,\ldots ,\mathbf{v }_d$ are unknown *p*-dimensional linearly independent column vectors, $d \ll p$, *g* is an unknown link function, $\varepsilon$ is a random error term with an arbitrary and unknown distribution, and $\varepsilon \perp \!\!\!\perp {{\mathbf{X}}}$, where $\perp \!\!\!\perp$ denotes statistical independence. Under model (1), the response *Y* depends on the *p*-dimensional predictor vector ${{\mathbf{X}}}$ only through the *d* linear combinations $\mathbf{v }^{\top }_1{{\mathbf{X}}},\ldots ,\mathbf{v }^{\top }_d{{\mathbf{X}}}$. Consequently, (1) is often expressed as $Y \perp \!\!\!\perp {{\mathbf{X}}}| (\mathbf{v }^{\top }_1{{\mathbf{X}}},\ldots ,\mathbf{v }^{\top }_d{{\mathbf{X}}})$. If we knew $\mathbf{v }_1,\ldots ,\mathbf{v }_d$, since $d \ll p$, estimation of the unknown link function *g* can be facilitated with the aid of any flexible nonparametric method. Therefore, the focus of dimension reduction methods via (1) is on estimating the vectors $\mathbf{v }_1,\ldots ,\mathbf{v }_d$ without any prior knowledge or assumption on *g*. The subspace span$(\mathbf{v }_1,\ldots ,\mathbf{v }_d)$ is called the *central subspace* and denoted by ${\mathcal {S}}_{Y|{{\mathbf{X}}}}$, where *d* is the smallest dimension such that (1) holds.

In the past two decades, a number of sufficient dimension reduction methods have been proposed to estimate $\mathbf{v }_1,\ldots ,\mathbf{v }_d$ under (1). The most widely used methods are perhaps the sliced inverse regression (SIR) by [1], and the sliced average variance estimation (SAVE) by [2]. Let ${\varvec{\Sigma }}= \text{cov}({{\mathbf{X}}})$, and denote $\mathbf{V } = (\mathbf{v }_1,\ldots ,\mathbf{v }_d) \in {\mathbb {R}}^{p \times d}$. In a pioneering paper, [1] established that the centered inverse regression, ${\mathbb {E}}({{\mathbf{X}}}|Y)-{\mathbb {E}}({{\mathbf{X}}})$, is contained in the linear subspace of ${\mathbb {R}}^p$ spanned by the vectors ${\varvec{\Sigma }}\mathbf{v }_1,\ldots ,{\varvec{\Sigma }}\mathbf{v }_d$, under model (1) and the linear conditional mean assumption $\big ($ i.e. ${\mathbb {E}}\big ({{\mathbf{X}}}|{\mathbf{V}}^{\top }{{\mathbf{X}}}\big )$ is a linear function of $\mathbf{V }^{\top }{{\mathbf{X}}}$ $\big )$. A direct consequence of this result is that the *kernel matrix* ${\mathbf{M}} = \text{cov} [ {\mathbb {E}}({{\mathbf{X}}}|Y)-{\mathbb {E}}({{\mathbf{X}}})]$ is degenerate in any direction ${\varvec{\Sigma }}$-orthogonal to the $\mathbf{v }_j$’s. Therefore, the eigenvectors corresponding to the *d* nonzero eigenvalues of ${\varvec{\Sigma }}^{-1} \mathbf{M }$ can be used as the estimators of $\mathbf{v }_1,\ldots ,\mathbf{v }_d$. A drawback of the sliced inverse regression is, since it only exploits the inverse first moment, ${\mathbb {E}}({{\mathbf{X}}}|Y)$, it yields degenerate directions if the model is symmetric about zero. To this end, [2] proposed the sliced average variance estimation that incorporates information in the inverse second moment. Since then, there have been a number of other proposals that exploit the moments of ${{\mathbf{X}}}|Y$. The recurring theme of these *inverse regression* methods is to construct a method-specific *kernel matrix* **M** degenerate in any direction ${\varvec{\Sigma }}$-orthogonal to the $\mathbf{v }_j$’s that span ${\mathcal {S}}_{Y|{{\mathbf{X}}}}$.

The aforementioned inverse regression methods for sufficient dimension reduction (SDR) yield the *d*-dimensional *sufficient predictors*, $\mathbf{v }^{\top }_1{{\mathbf{X}}},\ldots ,\mathbf{v }^{\top }_d{{\mathbf{X}}}$, that are linear combinations of all the original predictors. As a consequence, no variable selection is achieved. Hence, the results can be hard to interpret and the important variables may be difficult to identify. In addition, the estimation and prediction efficiency gain may be less than that possible with variable selection. To this end, methods that perform simultaneous dimension reduction and variable selection to construct a few *sufficient predictors* that are linear combinations of only the *important original predictors* have been proposed. An incomplete list of such methods include: the shrinkage sliced inverse regression introduced by [3]; the sparse sufficient dimension reduction method due to [4]; and the general shrinkage strategy for sparse inverse regression estimation proposed by [5]. A common limitation of these methods is that the variable selection procedure used is coordinate-dependent, in the sense that they introduce element-wise (coordinate-wise) sparsity as opposed to row-wise (predictor) sparsity on **V**. The element-wise sparsity approach is not desirable because we would like to deem a predictor unimportant based on its contribution across all dimension reduction vectors simultaneously. To address this problem, [6] proposed a coordinate-independent sparse estimation (CISE) method to obtain row-wise sparse estimates using the inverse regression methods. Due to the row-sparsity, CISE solutions are also orthogonal transformation invariant. That is, the estimated zero rows on **V** will also be estimated as zero even if the dimension reduction subspace was represented by **VO**, where **O** is any $d \times d$ orthogonal matrix. This is an attractive property since $\mathbf{v }_1,\ldots ,\mathbf{v }_d$ are often not unique, but the subspace spanned by these vectors is unique. However, although these methods perform variable selection, they are not applicable to the high-dimensional setting.

To this end, a number of recent papers have proposed sparse sufficient dimension reduction methods for the high-dimensional setting. These methods can be categorized into three approaches. The first category employs different types of regularizations to develop shrinkage based methods for simultaneous dimension reduction and variable selection. For instance, [7] proposed sparse ridge sliced inverse regression by introducing $\ell _1$ and $\ell _2$-regularization to the least squares formulation of sliced inverse regression [8] to achieve dimension reduction and variable selection simultaneously. Yu et al. [9] showed estimation consistency by adopting the Dantzig selector to solve the generalized eigenvalue problem formulation of SIR under sparse covariance assumptions. But their estimation follows the sequential estimation approach which yields coordinate-dependent sparse estimates. Wang et al. [10] re-cast SIR into a “pseudo” sparse reduced-rank regression problem and showed consistency in central subspace estimation. By constructing artificial response variables made up from top eigenvectors of the estimated conditional covariance matrix, [11] introduced the Lasso-SIR method to obtain sparse estimates of the SIR dimension reduction directions. More recently, [12] proposed a convex formulation for sparse SIR, and [13] proposed the sparse minimum discrepancy approach for simultaneous dimension reduction and variable selection that incorporates SIR, with extension to SAVE and the principal fitted components (PFC; [14, 15]). The second approach for high-dimensional SDR is the sequential SDR framework proposed by [16] and [17]. This framework yields simulataneous dimension reduction and variable selection via a sequential process that allows for $p > n$. It incorporates well-established SDR methods and has shown successful applications in high-dimensional data analysis. The third approach includes the thresholding-type procedures. The thresholding techniques have shown important applications for variable screening purposes in, for example, [18]. A promising diagonal thresholding screening SIR algorithm [19] was developed for sparse predictor covariance scenarios and the estimation consistency was established under high-dimensional setting. However, it does not yield sparse central subspace for variable selection. A concise review of the sparse sufficient dimension reduction literature can be found in [20].

Our work in this paper contributes to this body of literature by studying a convex formulation that produces simultaneous dimension reduction and variable selection. Our formulation can be interpreted as a version of a group Dantzig selector and falls under the first category of regularization based methods for high-dimensional sparse SDR. We minimize the sum of the block-$\ell _1$-norm of the row vectors that span the central subspace. Due to the row-sparse nature of the resulting estimator, our formulation leads to coordinate-independent sparse estimates - in the sense that the predictors selected by our method are independent of the basis matrix used to represent the central subspace. This is attractive as, often times, the central subspace is unique but the basis vectors that span it are not. Our proposed formulation is convex, and thus can be implemented using well established solvers such as the CVX toolbox in MATLAB. We provide readily available MATLAB codes for practitioners to use the proposed method.

Our work closely relates to the Lasso-SIR of [11] in the way it constructs artificial response matrix, but unlike our method, the estimated directions obtained by the Lasso-SIR [11] are not coordinate independent as the directions are estimated separately. Our work also relates to the convex formulation for sparse SIR of [12], in that both methods are based on convex optimization. However, the objective function in [12] is optimized over the $p \times p$ projection matrix, $\mathbf{VV} ^\top$, while our objective function is optimized over the $p \times d$ direction matrix **V**, $d \ll p$. If the number of predictors, *p*, is large, the method proposed in [12] is likely to be computationally more expensive than our method.

As is the case with most SDR methods, the sparse estimation method we propose in this paper can be used for regression (i.e. quantitative response) setting and classification problems (i.e. categorical response). Under model (1), [21] pointed out that linear discriminant analysis (LDA) is equivalent to SIR in the population. Cook and Yin [22] also further established the relationship between LDA and SIR, as well as quadratic discriminant analysis (QDA) and SAVE, and presented applications of (1) in discriminant analysis. These close connections also motivated us to explore the competitiveness of empirical results using our method with existing state-of-the-art generalizations of LDA for high-dimensional and multi-class classification problems.

*Organization*. The rest of the article is organized as follows. In “Method” section we describe the proposed method, and discuss its implementation. In “Continuous response” section we conduct extensive simulation studies to assess the performance of the proposed estimator and compare to other estimators in the literature. We apply our method to three omics datasets and demonstrate its use in practice in “Categorical response” section. We offer brief discussion in “Summary and conclusion” section.

*Notations*. For a vector $\mathbf{v } \in {\mathbb {R}}^p$, we define $\Vert \mathbf{v }\Vert _\infty = \max _{i=1,\ldots ,p}|v_i|$, $\Vert \mathbf{v }\Vert _1 = \sum _{i=1}^p |v_i|$, and $\Vert \mathbf{v }\Vert _2 = \sqrt{\sum _{i=1}^p v_i^2}$. For a matrix $\mathbf{M } \in {\mathbb {R}}^{n \times p}$ we define $\mathbf{m }_i$ to be its *i*th row, $\mathbf{m }_j$ to be its *j*th column, $\Vert \mathbf{M }\Vert _\infty = \max _{i=1,\ldots ,n}\Vert \mathbf{m }_i\Vert _1$, $\Vert \mathbf{M }\Vert _{\infty ,2} = \max _{i=1,\ldots ,n}\Vert \mathbf{m }_i\Vert _2$, and $\Vert \mathbf{M }\Vert _{\text{F}} = \sqrt{ \sum _{i=1}^n \sum _{j=1}^p m_{ij}^2}$.

## Method

As stated in the background section, let ${\mathcal {S}}_{Y|{{\mathbf{X}}}}$ denote the central subspace, and let ${\varvec{\Sigma }}= \text{cov}({{\mathbf{X}}})$. Define a population seed as any matrix ${\varvec{\Delta }}$ such that $\text{span}({\varvec{\Delta }}) \subseteq {\varvec{\Sigma }}{\mathcal {S}}_{Y|{{\mathbf{X}}}}$ and possibly $\text{span}({\varvec{\Delta }}) = {\varvec{\Sigma }}{\mathcal {S}}_{Y|{{\mathbf{X}}}}$. Here, we assume the *coverage condition*, $\text{span}({\varvec{\Delta }}) = {\varvec{\Sigma }}{\mathcal {S}}_{Y|{{\mathbf{X}}}}$, to hold. The coverage condition is commonly made in sufficient dimension reduction literature, and may be reasonable in many applications, see Cook and Ni [23] for discussion. Thus, if ${\varvec{\Sigma }}$ is invertible, a seed matrix can be used to obtain a matrix $\mathbf{V }$ whose columns span the central subspace by setting $\mathbf{V } = {\varvec{\Sigma }}^{-1}{\varvec{\Delta }}$. For example, for the ordinary least squares method, the $p \times 1$ covariance vector ${\varvec{\Delta }}= \text{cov}({{\mathbf{X}}}, Y)$ is the seed vector, and the central subspace can be obtained as the span of the least squares vector $\mathbf{V } = {\varvec{\Sigma }}^{-1}{\varvec{\Delta }}$, if $d=1$.

Let $\mathbf{V } \in {\mathbb {R}}^{p \times d}$ be a matrix such that span(**V**) = ${\mathcal {S}}_{Y|{{\mathbf{X}}}}$. In his pioneering sliced inverse regression estimation paper, under the linear conditional mean assumption (i.e. ${\mathbb {E}}({{\mathbf{X}}}|\mathbf{V }^{\top }{{\mathbf{X}}})$ is a linear function of the *d*-dimensional random vector $\mathbf{V }^{\top }{{\mathbf{X}}}$), [1] showed that

2 $$\begin{aligned} {\mathbb {E}}({{\mathbf{X}}}|Y=y)-{\mathbb {E}}({{\mathbf{X}}}) \in {\varvec{\Sigma }}{\mathcal {S}}_{Y|{{\mathbf{X}}}} \end{aligned}$$

for all *y*. The conditioning in (2) cannot be performed in practice unless *Y* is discrete, and standard practice with a continuous response is first to partition *Y* into *H* slices, indexed by $h = 1,\ldots , H$, and then average (2) over the values of *Y* in a slice [1]. This yields, $\psi _h \equiv {\mathbb {E}}\{{{\mathbf{X}}}|J_h(Y)=1\}-{\mathbb {E}}({{\mathbf{X}}}) \in {\varvec{\Sigma }}{\mathcal {S}}_{Y|{{\mathbf{X}}}}, h=1,\ldots ,H,$ where $J_h(Y)=1$ if *Y* is in slice *h* and $J_h(Y)=0$ otherwise. When the response is categorical, *H* is set to be the number of categories by construction, and when the response is continuous, *H* must satisfy $H \ge d$. Let ${\varvec{\Psi }}$ to be the $p \times H$ matrix ${\varvec{\Psi }}= (\psi _1,\ldots ,\psi _H)$. Then, it follows that ${\varvec{\Psi }}$ qualifies as a seed matrix. Furthermore, it follows that the SIR kernel matrix $\mathbf{M } = \text{cov}[{\mathbb {E}}({{\mathbf{X}}}|Y)-{\mathbb {E}}({{\mathbf{X}}})] = {\varvec{\Psi }}{\varvec{\Psi }}^{\top }$ qualifies as a seed matrix. Consequently, the sliced inverse regression estimation can be formulated as a generalized eigenvalue problem,

3 $$\begin{aligned} \mathbf{M }\mathbf{v }_j = \lambda _j {\varvec{\Sigma }}\mathbf{v }_j, \quad (j=1,\ldots , p) \end{aligned}$$

where $\lambda _j$’s are the eigenvalues of ${\varvec{\Sigma }}^{-1}\mathbf{M }$, and $\mathbf{v }_j$’s are the corresponding eigenvectors. The *d* eigenvectors, corresponding to the *d* nonzero eigenvalues span the central subspace.

Let $\big \{ \mathbf{x }_i^{\top },y_i \big \}_{i=1}^n$ denote an available *n* iid samples, $\widehat{\mathbf{M }}$ and ${\widehat{{\varvec{\Sigma }}}}$ be the sample estimates of **M** and ${\varvec{\Sigma }}$, respectively. That is, with

$$\begin{aligned} {\bar{\mathbf{x }}} = n^{-1}\sum _{i=1}^n \mathbf{x }_i, \quad \text{and}\quad {\bar{\mathbf{x }}}_h = n_h^{-1} \sum _{\{i: J_h(y_i)=1\}}^n \mathbf{x }_i \end{aligned}$$

we compute the estimates as

$$\begin{aligned} {\widehat{{\varvec{\Sigma }}}} = n^{-1} \sum _{i=1}^n (\mathbf{x }_i - {\bar{\mathbf{x }}}) (\mathbf{x }_i - \bar{\mathbf{x }})^\top , \quad \widehat{\mathbf{M }} = \sum _{h=1}^H \frac{n_h}{n}({\bar{\mathbf{x }}}_h - \mathbf{x })({\bar{\mathbf{x }}}_h - \mathbf{x })^\top . \end{aligned}$$

For simultaneous coordinate-independent sparse sliced inverse regression estimation, we propose the following optimization problem

4 $$\begin{aligned} {\widehat{\mathbf{V }}} = \min _{\mathbf{V }} \sum _{i=1}^p \Vert \mathbf{v }_i\Vert _2 \quad \text{subject to} \quad \Vert {\widehat{{\varvec{\Sigma }}}}^{-1/2}\widehat{\mathbf{M }} {\widehat{{\varvec{\Sigma }}}}^{-1/2} {\tilde{\mathbf{V }}} - \mathbf{V } {\tilde{\Lambda }}\Vert _{\infty } \le \tau , \end{aligned}$$

where $\mathbf{v }_i$, $i=1,\ldots ,p$, are the rows of **V**, ${\tilde{\Lambda }}$ is a diagonal matrix with the *d* nonzero eigenvalues of ${\widehat{{\varvec{\Sigma }}}}^{-1/2}\widehat{\mathbf{M }} {\widehat{{\varvec{\Sigma }}}}^{-1/2}$, $\tilde{\mathbf{V }}$ is a $p \times d$ matrix of the corresponding non-sparse eigenvectors, and $\tau > 0$ is a tuning parameter that controls the row-sparsity of ${\widehat{\mathbf{V }}}$. As $\tau$ increases, it leads to solutions ${\widehat{\mathbf{V }}} ({\tau })$ with more row-sparsity. Note again that the target basis matrix $\mathbf{V }$ is $p \times d$, and the $\Vert \mathbf{v }_i\Vert _2$’s in the objective function are defined row-wise, aggregated over the *p* rows corresponding to the *p* predictors. We could think of (4) as a group dantzig type formulation [24], where the group refers to a predictor’s contribution to the *d* dimension reduction vectors, and that the objective function is defined as a block-$\ell _1$-norm of the row vectors. The solution to (4) will not necessarily yield an orthogonal basis matrix ${\widehat{\mathbf{V }}}$. Nevertheless, we can obtain a sparse basis matrix via a Gram-Schmidt orthogonalization of the final estimate. The objective function is independent of the basis used to represent the span of $\mathbf{V }$, since for any orthogonal matrix $\mathbf{O }$, $\psi (\mathbf{V }) = \psi (\mathbf{VO} )$, where $\psi (\mathbf{V }) = \sum _{i=1}^p \Vert \mathbf{v }_i\Vert _2$, and the non-zero rows in **V** and **VO** are the same.

In the classical $p < n$ regime, we can obtain $\tilde{\mathbf{V }}$ by doing a singular value decomposition on ${\widehat{{\varvec{\Sigma }}}}^{-1/2}\widehat{\mathbf{M }} {\widehat{{\varvec{\Sigma }}}}^{-1/2}$. However, in the high dimensional setting, $p > n$, it is important for the performance of our proposed method via (4) that the estimate $\tilde{\mathbf{V }}$ be reasonable. If **M** is a $p \times p$ nonnegative definite matrix with rank(**M**) = $d \le p$, and ${\varvec{\Sigma }}$ is a $p \times p$ positive definite matrix, the true **V** satisfies $\mathbf{MV} = {\varvec{\Sigma }}\mathbf{V }\Lambda$. In the high dimensional setting, we assume **V** is *s*-sparse for some fixed *s*, and denote $supp(\mathbf{V }) = F = \{i: \Vert \mathbf{v }_i\Vert _2 \ne 0, i = 1,\ldots ,p\}$, where $|F| = s$ represent the number of relevant predictors. Let $\widehat{\mathbf{M }}$ and ${\widehat{{\varvec{\Sigma }}}}$ be sample estimates of **M** and ${\varvec{\Sigma }}$ that preserve the same definiteness as their population counterparts. Since $\widehat{\mathbf{M }}\tilde{\mathbf{V }} = {\widehat{{\varvec{\Sigma }}}}\tilde{\mathbf{V }}{\tilde{\Lambda }}$, we can write the constraint in (4) as $\big \Vert {\widehat{{\varvec{\Sigma }}}}\tilde{\mathbf{V }}-{\widehat{{\varvec{\Sigma }}}}\mathbf{V } \big \Vert _\infty \le \tau .$ In the high dimensional setting, we assume that $\tau = O\big (s\sqrt{\log p/n}\big )$, and thus $\tilde{\mathbf{V }}$ should satisfy $\big \Vert {\widehat{{\varvec{\Sigma }}}}\tilde{\mathbf{V }}-{\widehat{{\varvec{\Sigma }}}}\mathbf{V } \big \Vert _\infty \le \big (s\sqrt{\log p/n}\big ).$ In the next paragraph, we discuss an approach for obtaining ${\varvec{\Sigma }}^{-1}$ that yields $\tilde{\mathbf{V }}$.

*Estimation of* ${\varvec{\Sigma }}^{-1}$: When *p* is greater than *n*, ${\widehat{{\varvec{\Sigma }}}}$ is no longer invertible even when ${\varvec{\Sigma }}$ is nonsingular, and it is not possible to get a reasonable estimate ${\tilde{{{\mathbf{V}}}}}$. Therefore, we need a good estimate of ${\varvec{\Sigma }}^{-1}$ for $n < p$. Estimation of ${\varvec{\Sigma }}^{-1}$ has been extensively studied in the literature. In this work, we will simply adopt the constrained $\ell _1$ minimization for inverse covariance matrix estimation (CLIME) method proposed by [25]. Denote ${\varvec{\Omega }}:= {\varvec{\Sigma }}^{-1}$. Given a tuning parameter $\lambda _{1n}$, the CLIME based estimate ${\widehat{{\varvec{\Omega }}}}$ is a solution to the following optimization problem:

$$\begin{aligned} \min _{{\varvec{\Omega }}} \Vert {\varvec{\Omega }}\Vert _1\quad \text{subject to}\quad \Vert {\widehat{{\varvec{\Sigma }}}}{\varvec{\Omega }}- \mathbf{I }\Vert _\infty \le \lambda _{1n}. \end{aligned}$$

The above solution ${\widehat{{\varvec{\Omega }}}}$ is not symmetric in general. To obtain a symmetric estimate, the CLIME estimator ${\widehat{{\varvec{\Omega }}}}_s$ is defined as ${\widehat{{\varvec{\Omega }}}} := ({\widehat{{\varvec{\Omega }}}}_{s,k,l})$ where

$$\begin{aligned} {\widehat{{\varvec{\Omega }}}}_{s,k,l} = {\widehat{{\varvec{\Omega }}}}_{s,l,k} = {\widehat{{\varvec{\Omega }}}}_{k,l}I(|{\widehat{{\varvec{\Omega }}}}_{k,l}| \le |{\widehat{{\varvec{\Omega }}}}_{l,k}|) + {\widehat{{\varvec{\Omega }}}}_{l,k}I(|{\widehat{{\varvec{\Omega }}}}_{k,l}| > |{\widehat{{\varvec{\Omega }}}}_{l,k}| ). \end{aligned}$$

In other words, we take the one with smaller magnitude between ${\widehat{{\varvec{\Omega }}}}_{k,l}$ and ${\widehat{{\varvec{\Omega }}}}_{l,k}$. The resulting estimate ${\widehat{{\varvec{\Omega }}}}_s$ is symmetric and, more importantly, positive definite with high probability. By assuming that the covariates have exponential type tails and $\lambda _{1n} = C_1\sqrt{\log p/n}$ for some generic constant $C_1$, [25] show that

$$\begin{aligned} \Vert {\widehat{{\varvec{\Omega }}}} - {\varvec{\Omega }}\Vert _\infty = O_p(M^2 s_1 \sqrt{\log p /n}), \end{aligned}$$

holds uniformly for

$$\begin{aligned} {\varvec{\Omega }}\in \left\{ {\varvec{\Omega }}: {\varvec{\Omega }}> 0, \Vert {\varvec{\Omega }}\Vert _1 \le M \text{ and } \max _{1 \le k \le p} \sum _{l=1}^p |{\varvec{\Omega }}_{k,l}|^q \le s_1 \right\} . \end{aligned}$$

for $0 \le q < 1$. Note that the special case of $q=0$ is a class of $s_1$-sparse matrices.

Given an estimate ${\widehat{{\varvec{\Sigma }}}}^{-1}$, we implement the optimization problem in (4) using the CVX, an efficient MATLAB package for specifying and solving convex optimization problems [26, 27].

### Selection of the tuning parameter

The tuning parameter $\tau$ in (4) controls the level of sparsity and needs to be selected. Note that when $\tau >\Vert {\widehat{{\varvec{\Sigma }}}}^{-1/2}\widehat{\mathbf{M }}{\widehat{{\varvec{\Sigma }}}}^{-1/2} \tilde{\mathbf{V }}\Vert _\infty$, the optimization problem (4) yields a trivial solution, giving us an upper bound for $\tau$. We choose the optimal $\tau$ from the range $\big (0,\Vert {\widehat{{\varvec{\Sigma }}}}^{-1/2}\widehat{\mathbf{M }}{\widehat{{\varvec{\Sigma }}}}^{-1/2} \tilde{\mathbf{V }}\Vert _\infty \big )$ using *K*-fold cross validation (CV). More specifically, for the categorical response case, we randomly group the observations of $\mathbf{X }$ into *K* roughly equal-sized groups, denoted as $\mathbf{X }^{1},\ldots ,\mathbf{X }^{K}$. For each $k=1,\ldots ,K$, let $\mathbf{X }^{-k}$ be the input data matrix leaving out $\mathbf{X }^{k}$. Let $\mathbf{y }^{k}$ and $\mathbf{y }^{-k}$ be the corresponding response vectors. We apply the proposed methods on $\mathbf{X }^{-k}$ to derive basis matrices $\widehat{\mathbf{V }}^{-k}_{d}(\tau ), d={\textit{rank}}(\widehat{\mathbf{M }})$, and the data $\mathbf{X }^{k}$ are then projected onto $\widehat{\mathbf{V }}^{-k}_{d}(\tau )$ to obtain discriminant scores $\mathbf{U }_{d}(\tau _{n})= \mathbf{X }^{k}\widehat{\mathbf{V }}^{-k}_{d}(\tau )$, and classification of $\mathbf{X }^{k}$ is performed using the nearest centroid method to obtain predicted response $\mathbf{y }^{k}_{pred}(\tau )$. We calculate the *K*-fold CV misclassification rate as

5 $$\begin{aligned} CV(\tau )=\frac{1}{K}\sum _{k=1}^{K}\frac{\# \left( \mathbf{y }^{k}_{pred}(\tau ) \ne \mathbf{y }^{k} \right) }{n_{k}} \end{aligned}$$

where $n_{k}$ is the number of observations in $\mathbf{X }^{k}$. We do this for each $\tau$ and select the optimal tuning parameter as $\tau _{opt}=\min _{\tau _{n}}\{CV(\tau )\}$.

For the continuous response case, we adopted the following information criteria method suggested in [9]. Define the average squared residuals as

$$\begin{aligned} G(\tau ) = \frac{\text{tr} \{ \widehat{\mathbf{V }}^{\top }(\tau ){\widehat{{\varvec{\Sigma }}}}\widehat{\mathbf{V }}(\tau ) - \widehat{\mathbf{V }}^{\top }(\tau )\widehat{\mathbf{M }}\widehat{\mathbf{V }}(\tau ) \} }{\text{tr}\{\widehat{\mathbf{V }}^{\top }(\tau )\widehat{\mathbf{M }}\widehat{\mathbf{V }}(\tau )\}} \end{aligned}$$

where $\widehat{\mathbf{V }}(\tau )$ is the estimate of **V** obtained from (4) with a given $\tau$. Denote the number of nonzero rows of $\widehat{\mathbf{V }}(\tau )$ by $s(\tau )$. We select optimal tuning parameter as

6 $$\begin{aligned} \tau _{opt} = \min _{\tau } \text{BIC}(\tau ) = \min _{\tau } \{ n \log (G(\tau )) + \log (n)s(\tau )\}. \end{aligned}$$

## Results

### Simulation studies

In this section, we conduct extensive simulations to assess the performance of the proposed method and compare it with other competing methods in the literature. We consider both continuous and categorical response cases.

#### Continuous response

Here we simulate models with continuous response variable. We assess estimation accuracy and variable selection selection accuracy. To assess variable selection performance, we report the true positive rate (TPR) and false positive rate (FPR). TPR is the proportion of truly important variables with estimated nonzero corresponding rows, and FPR is the proportion of unimportant variables with estimated nonzero corresponding rows. A method that does well in variable selection will have a TPR close to one, and an FPR close to zero. For estimation and prediction performance, we report the correlation coefficient between the true sufficient predictor ($\mathbf{v }^{\top }{{\mathbf{X}}})$ and estimated sufficient predictor ($\widehat{\mathbf{v }}^{\top }{{\mathbf{X}}})$. For the two-dimensional Model (9), we report the average of these two correlation coefficients.

We simulate data using the three regression models below, adopted from [12]. We adopt the following simulation settings from [12]. We generate the predictor vector ${{\mathbf{X}}}$ from $N(0,{\varvec{\Sigma }})$, where ${\varvec{\Sigma }}_{ij} = 0.5^{|i-j|}$ and $\varepsilon \sim N(0,1)$. We compare the performance of our proposed method with the performance of [10, 12, 16] and [7]. A linear regression model with three active predictors:

7 $$\begin{aligned} y = (x_1 + x_2 + x_3)/\sqrt{3} + 2\varepsilon , \end{aligned}$$

where the central subspace is spanned by the direction $\mathbf{v }_1= (1,1,1,0_{p-3})^{\top }$, and $d=1$.

A single-index nonlinear regression model with three active predictors:

8 $$\begin{aligned} y = 1 + \exp \{(x_1 + x_2 + x_3)/\sqrt{3}\} + \varepsilon , \end{aligned}$$

where the central subspace is spanned by the direction $\mathbf{v }_1 = (1,1,1,0_{p-3})^{\top }$, and $d=1$. This model was also studied in [16].

A multi-index nonlinear regression model with five active predictors:

9 $$\begin{aligned} y = \frac{x_1 + x_2 + x_3}{0.5 + (x_4 + x_5 + 1.5)^2} + 0.1\varepsilon , \end{aligned}$$

where the central subspace is spanned by the directions $\mathbf{v }_1 = (1,1,1,0_{p-3})^{\top }$, and $\mathbf{v }_2 = (0,0,0,1,1,0_{p-5})^{\top }$, and $d=2$. This model form has been used extensively in the sufficient dimension reduction literature, see for instance [1].

#### Summary of Simulation Results

Table 1 presents the results for Models (7)–(9). The results show that our proposed method performs very competitively against recent proposals for sparse sliced inverse regression for high-dimensional data [7, 10, 12, 16]. More specifically, we make the following observations. In the classical setting ($n = 200$, $p=150$), while all the methods yield very good results, our method yields the best results, followed by [12]. In the high-dimensional setting ($n = 100$, $p=150$), for the single-index models (Models (7) and (8)), our method yields the best results in terms of TPR and correlation. In terms of FPR, [10] yields the best results. However, our method also yields reasonable FPR values, comparable with the rest of the methods. Model (9) is multi-index model and the performances of all the methods are inferior to their performance in the single-index case. Nevertheless, our method still yields competitive results with the other methods, with the exception of the [16] methods that performs very well in terms of variable selection, but struggles overall in terms of the correlation. However, the reported results for [16] are the best results after considering multiple tuning parameters, unlike the other methods use data adaptive tuning parameter selection approach. In summary, our proposed method yields the best overall performance across the three Models and two settings (classical and high-dimensional setting).

**Table 1:** Simulation results for Models (7)–(9)

		$\boldsymbol{n}=\textbf{100},\,\boldsymbol{p}=\textbf{150}$			$\boldsymbol{n}=\textbf{200},\,\boldsymbol{p}=\textbf{150}$		
		Model (7)	Model (8)	Model (9)	Model (7)	Model (8)	Model (9)
Proposed method	TPR	100 (2.3)	99 (0.1)	84 (1.8)	100 (0.0)	100 (0.0)	91 (1.4)
	FPR	6.8 (0.5)	11 (0.1)	7.5 (8.0)	0.0 (0.0)	0.0 (0.0)	1.0 (0.1)
	Corr	98 (2.3)	94 (0.1)	72 (0.2)	99 (0.0)	99 (1.0)	97 (2.0)
[12]	TPR	96 (1.0)	94 (1.2)	91 (1.1)	98(0.5)	99 (0.5)	99 (2.5)
	FPR	6.0 (0.9)	3.6 (0.7)	7.4 (0.1)	3.4 (0.4)	1.1 (0.2)	2.5 (0.3)
	Corr	88 (0.9)	86 (1.1)	74 (1.1)	91 (0.5)	92 (0.5)	79 (0.6)
[16]	TPR	95 (0.9)	100 (0.0)	100 (0.6)	100 (0.0)	100 (0.0)	100 (0.0)
	FPR	4.9 (0.1)	4.8 (0.1)	3.5 (0.1)	5.9 (0.2)	6.7 (0.3)	4.5 (0.2)
	Corr	59 (1.1)	88 (0.5)	79 (0.6)	79 (0.6)	94 (0.2)	87(0.5)
[7]	TPR	98 (0.1)	98 (0.1)	98 (0.1)	99 (0.1)	99 (0.1)	98 (0.1)
	FPR	8.3 (1.2)	3.8 (0.8)	23 (1.1)	1.2 (0.4)	0.3 (0.2)	20 (1.1)
	Corr	84 (0.9)	89 (0.6)	63 (0.7)	94 (0.4)	96 (0.3)	70 (0.5)
[10]	TPR	89 (1.5)	94 (1.2)	80 (1.2)	98(1.0)	99 (0.7)	96 (0.6)
	FPR	0.6 (0.1)	0.6 (0.1)	0.2 (0.1)	0.3 (0.1)	0.3 (0.1)	0.1 (0.1)
	Corr	82 (1.4)	85 (1.3)	70 (1.1)	91 (1.1)	93 (1.0)	84 (0.7)

Corr is the correlation coefficient between the true and estimated sufficient predictors; TPR is the true positive rate; FPR is the false positive rate. The mean (standard error), averaged over 200 independent replications, are reported. All entries are multiplied by 100

#### Categorical response

Here we conduct simulations for categorical response. We assess estimation accuracy of the central (discriminant) subspace, prediction accuracy after dimension reduction, and variable selection accuracy. We assess estimation accuracy using the Frobenius norm of the difference between the projection matrices of the true and estimated discriminant subspaces. More specifically, let $\widehat{\mathbf{V }}$ denote the estimate of $\mathbf{V }$. We measure closeness of $\widehat{\mathbf{V }}$ to $\mathbf{V }$ by

10 $$\begin{aligned} \Delta (\widehat{\mathbf{V }},\mathbf{V })=\Vert \widehat{\mathbf{V }}(\widehat{\mathbf{V }}^\top \widehat{\mathbf{V }})^{-1}\widehat{\mathbf{V }}^\top -\mathbf{V }(\mathbf{V }^\top \mathbf{V })^{-1}\mathbf{V }^\top \Vert _{\text{F}}, \end{aligned}$$

where $\Vert .\Vert _{\text{F}}$ denotes the Frobenius norm. Smaller values of this distance metric indicate a better estimate. We report TPR and FPR values for variable selection performance assessment. To assess the prediction performance, we report the generalization (test) misclassification rate (MSR).

We compare the performances of our method with three competing methods. The first competing method is the MGSDA by [28]. Like our proposed methods, MGSDA yields row-sparse linear discriminant analysis vectors for a multi-class classification problem. We implement MGSDA using the MGSDA package in R. The second competing method is the multi-class sparse discriminant analysis (MSDA) method by [29]. This method also imposes row-sparsity. For the binary classification case, MSDA reduces to the linear programming discriminant analysis (LPD) method by [30]. We implement MSDA using the MSDA package in R. The third competing method is the penalized linear discriminant analysis (PLDA) of [31]. Unlike the other methods mentioned above, for the multi-class setting, PLDA estimates the discriminant vectors in a sequential fashion starting with the first discriminant vector $\mathbf{v }_1$, with subsequent $\mathbf{v }_j$ found subject to orthogonality constraints. Sparsity is achieved by imposing the $\ell _1$-norm penalty to each of the vectors. Therefore, PLDA yields sparse estimates that are not necessarily coordinate-independent, and generally this method selects more predictors. We implement PLDA using the penalizedLDA package in R. The corresponding optimal tuning parameters for all the methods are chosen via fivefold cross-validation to minimize test misclassification rate. In the sequel, CISESIR, and CISELDA represent our proposed method with the SIR and LDA matrices, respectively. We simulate three models as follows.

*Model 1*

We simulate a three class classification problem. The input matrix ${{\mathbf{X}}}\in {\mathbb {R}}^{p \times n}=[{{\mathbf{X}}}_{1}, {{\mathbf{X}}}_{2},{{\mathbf{X}}}_{3}]$ with the true covariance matrix is

$$\begin{aligned} {\varvec{\Sigma }}=\left( \begin{array}{cc} {\tilde{{\varvec{\Sigma }}}} &{}\quad \mathbf{0 } \\ \mathbf{0 } &{}\quad \mathbf{I }_{p-s} \end{array} \right) , \end{aligned}$$

where ${\tilde{{\varvec{\Sigma }}}}$ is the covariance structure for signal variables, which we take to be ${\tilde{{\varvec{\Sigma }}}} = \rho \mathbf{J } + (1-\rho )\mathbf{I }$, **I** is the identity matrix and $\mathbf{J }$ is a matrix with all entries equal to one. We set $n_{k}=30$, for a total of 90 observations, and generate ${{\mathbf{X}}}_k$ from $\text{N}({\varvec{\mu }}_k,{\varvec{\Sigma }})$, where we take ${\varvec{\mu }}_1 = \mathbf{0 }$, ${\varvec{\mu }}_2 = (1,\ldots ,1,0,\ldots ,0)$ with only the first ten entries nonzero, and ${\varvec{\mu }}_3 = (0,\ldots ,0,-2,\ldots ,-2,0,\ldots ,0)$ with entries 11–20 nonzero. The true discriminant vectors $\mathbf{v }_1$ and $\mathbf{v }_2$ are the eigenvectors of ${\varvec{\Sigma }}^{-1}\mathbf{M }$ corresponding to its two nonzero eigenvalues, where $\mathbf{M }$ is the true between-class covariance matrix. We report results for $p = 50, 500, 1000$. The number of signal variables in this model is $s=20$, which is the number of nonzero rows in the discriminant space: $\mathbf{V } = (\mathbf{v }_1, \mathbf{v }_2)$.

*Model 2*

In model 1 we simulated a case where the within class covariances are the same across the three classes. In this model, we consider the scenario where the classes differ not only through their means, but also their covariances. The covariance matrices for the three classes are given as follows: for class 1, the covariance matrix has the same form as in model 1 with $\rho =0.9$; for class 2, the covariance matrix has entries ${\varvec{\Sigma }}_{ij} = 0.5^{|i-j|}$; for class 3, the covariance matrix is the identity matrix, $\mathbf{I }_p$.

*Model 3*

In models 1 and 2, we simulated data from the inverse regression setup, ${{\mathbf{X}}}| Y$. In this model, we simulate data from forward regression $Y|{{\mathbf{X}}}$. More specifically, we simulate ${{\mathbf{X}}}\sim N(\mathbf{0 },{\varvec{\Sigma }})$ and generate *y* using the logistic regression model:

11 $$\begin{aligned} Y \sim {\textit{Bernoulli}}(p) \quad {\text{where}} \quad p = \frac{e^{\mathbf{v }^{\top }{{\mathbf{X}}}}}{1 + e^{\mathbf{v }^{\top }{{\mathbf{X}}}}}. \end{aligned}$$

We keep the covariance matrix structure to be the same as in model 1, and we generate the nonzero coefficients of $\mathbf{v }$ from *U*(0.8, 1). The number of nonzero coefficients are set to 10 in this example. In both models 1 and 2, the discriminant subspace of interest was two dimensional. However, in this model, it is one dimensional, the space spanned by $\mathbf{v }$. Note also that while models 1 and 2 are a three-group problem, model 3 is a binary classification problem.

***Summary of Simulation Results:***The results for model 1 are reported in Table 2. For this model, we observe that the classification performances of all the methods are comparable. In terms of variable selection, we see that PLDA has the highest TPR across all settings, with MGSDA and MSDA yielding the lowest TPRs. Both our methods, CISESIR and CISELDA, yield comparable variable selection performance, with CISELDA performing slightly better in TPR and CISESIR performing slightly better in FPR. Both our methods also perform better than the competing methods when the correlation structure among the predictors is stronger $(\rho =0.9)$. More specifically, when $\rho =0.9$, MGSDA and MSDA suffer and yield poor results, and PLDA generally selects more variables and yields higher FPR. Notice also that the performances of our methods improve with increase in *p*. Overall, we observe that our methods yield competitive estimation, classification, and variable selection performances, and generally yield lower FPRs. The results for model 2 are reported in Table 3. For this model, CISELDA and PLDA are the best performers in terms of classification accuracy, while MSDA, CISESIR and CISELDA are the best performers in terms of variable selection. Again, MGSDA performs the worst among all the methods. Notice that the setting for this model is such that the class-level predictor covariance matrices are different. These results show that our methods are also robust to the LDA assumption of constant within-group covariance matrix. Table 4 reports the results for model 3. The results for this model are similar to the results for model 1, confirming that the performance of our methods in binary classification problem resembles their performances in the three class problem (Table 2).

**Table 2:** Simulation results for model 1

Model 1						
($\rho$, *p*)		CISESIR	CISELDA	MGSDA	PLDA	MSDA
(0.5, 50)	$\Delta$	0.761	0.821	1.346	0.251	1.328
	MSR	0.128	0.127	0.137	0.125	0.137
	TPR	0.949	0.760	0.775	1.000	0.860
	FPR	0.100	0.227	0.101	0.225	0.264
(0.5, 500)	$\Delta$	0.797	0.888	1.374	0.455	1.315
	MSR	0.132	0.128	0.139	0.125	0.138
	TPR	0.897	0.985	0.733	1.000	0.810
	FPR	0.052	0.076	0.010	0.011	0.011
(0.5, 1000)	$\Delta$	0.632	0.604	1.384	0.406	1.307
	MSR	0.129	0.125	0.139	0.129	0.135
	TPR	0.932	0.999	0.739	1.000	0.794
	FPR	0.026	0.066	0.007	0.176	0.005
(0.9, 50)	$\Delta$	1.070	1.031	1.714	0.140	1.672
	MSR	0.209	0.207	0.213	0.206	0.215
	TPR	0.835	0.925	0.368	1.000	0.481
	FPR	0.140	0.214	0.037	0.254	0.164
(0.9, 500)	$\Delta$	0.925	1.086	1.730	0.409	1.703
	MSR	0.216	0.215	0.217	0.209	0.213
	TPR	0.828	0.998	0.376	1.000	0.399
	FPR	0.067	0.160	0.015	0.401	0.007
(0.9, 1000)	$\Delta$	0.588	0.663	1.047	0.289	1.680
	MSR	0.210	0.209	0.214	0.206	0.213
	TPR	0.942	1.000	0.393	1.000	0.427
	FPR	0.056	0.072	0.004	0.153	0.005

$\Delta$ is as defined in (10); TPR is the true positive rate; FPR is the false positive rate; MSR is the misclassification rate over a test set of 900 observations. Note again, TPR and FPR are with respect to variable selection. The reported numbers are averages over 50 repetitions

**Table 3:** Simulation results for model 2

Model 2						
*p*		CISESIR	CISELDA	MGSDA	PLDA	MSDA
50	$\Delta$	0.632	1.032	1.701	0.142	1.108
	MSR	0.101	0.048	0.122	0.037	0.104
	TPR	0.972	0.625	0.252	0.692	0.956
	FPR	0.075	0.200	0.072	0.231	0.248
500	$\Delta$	0.869	1.114	1.726	0.396	1.112
	MSR	0.111	0.053	0.123	0.040	0.104
	TPR	0.844	0.675	0.240	0.7612	0.916
	FPR	0.020	0.195	0.009	0.3824	0.021
1000	$\Delta$	0.786	0.711	1.708	0.366	1.100
	MSR	0.114	0.040	0.121	0.037	0.102
	TPR	0.851	0.644	0.241	0.694	0.922
	FPR	0.017	0.089	0.004	0.227	0.010

$\Delta$ is as defined in (10); TPR is the true positive rate; FPR is the false positive rate; MSR is the misclassification rate over a test set of 900 observations. Note again, TPR and FPR are with respect to variable selection. The reported numbers are averages over 50 repetitions

**Table 4:** Simulation results for model 3

Model 3						
($\rho$, *p*)		CISESIR	CISELDA	MGSDA	PLDA	MSDA
(0.5, 50)	$\Delta$	0.590	0.489	0.690	0.215	0.677
	MSR	0.208	0.086	0.111	0.080	0.100
	TPR	0.960	0.994	0.956	0.998	0.950
	FPR	0.357	0.102	0.090	0.204	0.118
(0.5, 500)	$\Delta$	0.438	0.536	0.746	0.241	0.683
	MSR	0.091	0.092	0.122	0.085	0.100
	TPR	0.978	0.990	0.924	1.000	0.942
	FPR	0.030	0.017	0.013	0.076	0.011
(0.5, 1000)	$\Delta$	0.322	0.302	0.751	0.218	0.749
	MSR	0.080	0.081	0.119	0.076	0.096
	TPR	0.994	1.000	0.922	1.000	0.900
	FPR	0.019	0.014	0.010	0.023	0.008
(0.9, 50)	$\Delta$	0.514	0.602	1.050	0.126	0.833
	MSR	0.070	0.069	0.097	0.067	0.077
	TPR	0.920	0.970	0.660	1.000	0.874
	FPR	0.292	0.082	0.077	0.335	0.059
(0.9, 500)	$\Delta$	0.273	0.390	1.048	0.229	0.814
	MSR	0.064	0.069	0.095	0.067	0.073
	TPR	0.988	1.000	0.638	1.000	0.868
	FPR	0.021	0.033	0.009	0.291	0.004
(0.9, 1000)	$\Delta$	0.492	0.228	1.047	0.185	0.772
	MSR	0.068	0.065	0.096	0.064	0.066
	TPR	1.000	1.000	0.652	1.000	0.850
	FPR	0.033	0.017	0.003	0.131	0.006

$\Delta$ is as defined in (10); TPR is the true positive rate; FPR is the false positive rate; MSR is the misclassification rate over a test set of 900 observations. Note again, TPR and FPR are with respect to variable selection. The reported numbers are averages over 50 repetitions

In summary, our simulation results show that our methods (CISESIR and CISELDA) yield competitive estimation, classification and variable selection performance. Our methods are among the best performing in terms of FPR in all settings. PLSD generally yields the highest TPR and FPR values because it selects more variables. This is not surprising since PLSD induces penalties to each of the dimension reduction (discriminant) vectors separately. Moreover, CISESIR and CISELDA yield the best overall variable selection performance in model 2 where the within group predictor covariance structures differ.

### Applications

#### Depression study

Metabolomics data on major depressive disorder (MDD) were obtained from the Metabolomics Workbench (see Data Availability Statement). In the original study, human cerebrospinal fluid and plasma samples were collected from patients diagnosed with MDD and control subjects matched on age and gender, and an untargeted metabolomics profiling was conducted on these samples. There were 158 metabolites on $n=48$ control patients and $n=46$ patients diagnosed with depression. Our goal in this study is to apply the proposed and existing competing methods to identify metabolites that optimally discriminate patients with MDD from patients without MDD.

We pre-process the data by eliminating metabolites with coefficient of variation greater than 50%—which leaves us with 103 metabolites. As is commonly done in metabolomics data analysis, we log2 transform each feature, and normalize each feature to have mean 0 and variance 1. Then, we randomly split the dataset into two-third training set and one-third test set. A stratified sampling scheme is applied to preserve the original proportions of samples in each group. We select the optimal tuning parameter that minimizes the average misclassification rate using fivefold cross-validation on the training set. We then apply the methods with the selected optimal tuning parameters to the test set to obtain an estimate of the generalization misclassification rate. We repeat the foregoing analysis scheme 50 times and obtain average misclassification rates and number of variables selected.

The average test misclassification rates, sensitivities, and specificities, along with their standard errors, obtained from the 50 splits are reported in Table 5. We see that the average test error for the proposed methods are comparable to that of MGSDA and MSDA, but are better than that of PLDA. MGSDA identifies fewer predictors, which agrees with the simulation results where it had high specificities and low sensitivities. For differentiating MDD patients from healthy controls, the proposed methods showed high sensitivity (CISELDA: 80.37, CISESIR: 84.62) and moderate to high specificities (CISELDA: 83.20, CISESIR: 72.27). Of the competing methods, PLDA had low specificity. Of note, these sensitivies and specificities were obtained comparing the observed class from the test data with the predicted class obtained using nearest centroid, and averaging over 50 data splits.

**Table 5:** Average misclassification rates and number of variables selected for the depression study

Method	Mean test error	Mean	Mean	Selected metabolites (SE)
		Sensitivity (%)	Specificity (%)	${\widehat{{\varvec{\beta }}}}$
CISESIR	0.1955 (0.0150)	84.62 (2.63)	72.27 (3.71)	40.540 (2.634)
CISELDA	0.1659 (0.0095)	80.37 (2.32)	83.20 (2.32)	26.280 (1.828)
MGSDA	0.1639 (0.0093)	84.62 (1.36)	81.47 (2.02)	16.129 (0.772)
PLDA	0.2484 (0.0093)	89.62 (0.97)	59.73 (2.36)	78.200 (2.797)
MSDA	0.1458 (0.0068)	84.75 (1.20)	86.13 (1.50)	58.480 (3.770)

Averages are over 50 repetitions of randomly splitting the data into training (63 observations) and testing (31 observations). Reported average error rates are obtained from the test sets

**Fig. 1:**
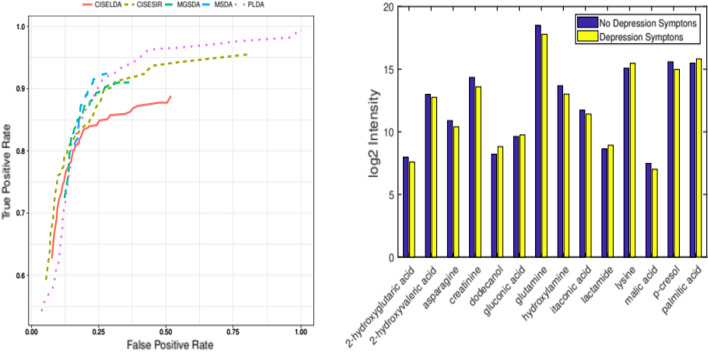
Left panel: ROC curve for the depression study data. Average AUC for CISELDA, CISESIR, MGSDA, MSDA, and PLDA are respectively 0.89, 0.94,0.91, 0.93, and 0.92. Right panel: Bar graphs of the log2 intensities for metabolites identified by CISELDA

We also investigate the metabolites identified by the proposed methods and how they may relate to depression. Here, we consider only metabolites that are selected all 50 times, a potential indication of the ability of these metabolites to contribute most to the differentiation of subjects with and without depression symptoms. When this was used, 14 metabolites (2-hydroxyglutaric acid, 2-hydroxyvaleric acid, asparagine, creatinine, dodecanol, gluconic acid, glutamine, hydroxylamine, itaconic acid, lactamide, lysine, malic acid, palmitic acid, and p-cresol) were selected by CISELDA for the separation between MDD and healthy controls, 12 metabolites (2-hydroxyglutaric acid, 2-hydroxyvaleric acid, asparagine, creatinine, dodecanol, glutamine, hydroxylamine, itaconic acid, lactamide, malic acid, palmitic acid, and p-cresol) where selected by CISESIR, 8 metabolites (asparagine creatinine, dodecanol, glutamine, lactamide, malic acid, palmitic acid, and p-cresol ) were selected by MGSDA, 10 metabolites (asparagine, creatinine, dodecanol, fructose, glutamine, hydroxylamine, lactamide, oxoproline, palmitic acid, and p-cresol) were selected by MSDA, and 90 meatabolites were selected by PLDA, of which CISELDA is a subset. Note that the 8 metabolites identified by MGSDA are subsets of CISESIR and CISESLDA. We report the log2 transformed intensity data for the metabolites identified by CISELDA for patients with depression symptoms compared to patients with no depression symptoms (right panel of Fig. 1).

Some of the metabolite biomarker candidates identified by our methods have been suggested to be depression-related compounds. For example, glutamine, which was significantly reduced for patients in our data - confirming other studies reporting that depressed patients had reduced levels of glutamine/glutamate [32], is suggested to be implicated in the pathophysiologic mechanisms of MDD [33, 34].

We also conduct pathway enrichment analaysis using MetaboAnalyst 3.3 for possible connections between these metabolites (http://www.metaboanalyst.ca/faces/ModuleView.xhtml). With a false discovery rate of 0.05, CISELDA, CISESIR, and MSDA identified the nitrogen metabolic pathway to be significantly enriched with three metabolites (Hydroxylamine, Glutamine, and Asparagine) in our list belonging to this pathway. Meanwhile, no pathway reached the FDR threshold for MGSDA. On the other hand, 8 pathways including the nitrogen pathway reached FDR threshold for the candidate metabolites identified by PLDA; this is not surprising since PLDA identified more metabolites. The nitrogen pathway plays an important role in the metabolism of nitrogen into other compounds that are essential for human survival.

#### RNA-seq data

In this example, we demonstrate the ability of the methods to identify features for discriminative purposes when there are more than two groups and when the number of variables is high. Advances and improvements in technology and decreasing cost of next-generation sequencing have made RNA sequencing (RNA-seq) a widely used method for gene expression studies. We used RNA-seq data on Drosophila Melanogaster (Fly) [35] downloaded from ReCount database [36]. Features with more than half their values being zero were filtered out. The remaining features with zero values were truncated at 0.5 and the data were then log-transformed. We filtered out features with low variances, resulting in $p=12,046$ dimensions. Finally, the data were normalized to have equal medians for each sample, and mean zero and unit variance for each feature. There were four fly classes: Class 1 consisted of all embryos; Class 2 consisted of all larvae; Class 3 consisted of all white prepupae; and Class 4 consisted of all adult flies. The data set consists of a total of $n=147$ samples. We split the data to 99 training set and 48 test set proportionately. The rest of the analysis was carried out similarly to the depression example.

**Fig. 2:**
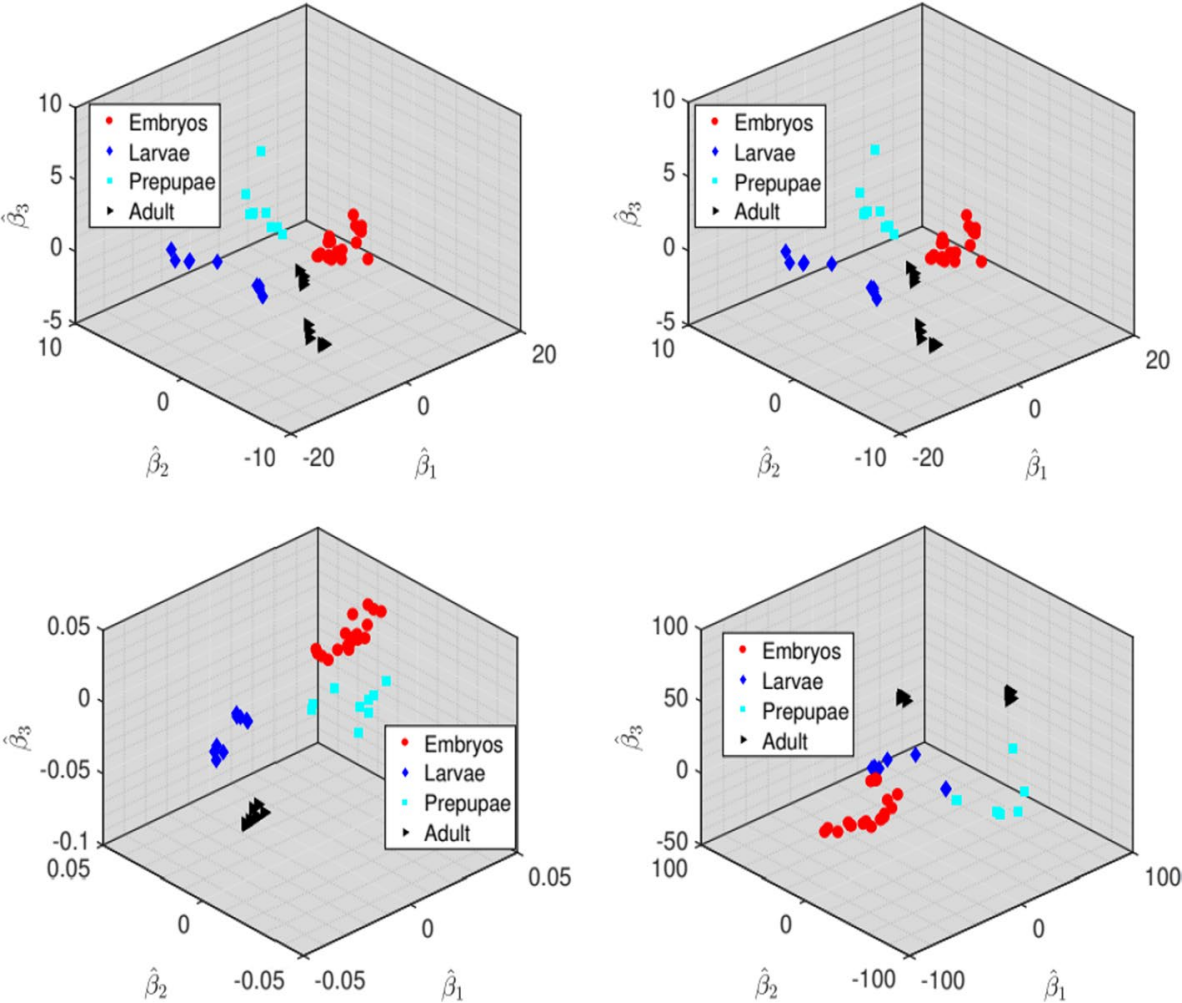
RNA-seq test set projected onto estimated discriminant vectors. Top-right panel: CISESIR; Top-left panel: CISELDA; Bottom-right panel: MGSDA; Bottom-left panel: PLDA

In Table 6 we report the classification performance in terms of average test misclassification rates. The proposed methods are competitive achieving similar or better classification accuracy when compared to the competing methods. In terms of variable selection, it is noticeable that MGSDA is most sparse, with PLDA being least sparse. This result is consistent with the simulation results where MGSDA had high specificities and low sensitivities. Figure 2 is a visual representation of one random split of the testing data projected onto the estimated sparse discriminant subspaces. It can be observed that the classes are well separated. MSDA took too long to run for this data. Therefore, the results for this method are not reported.

**Table 6:** Average misclassification rates and number of variables selected for the RNA-seq study

Method	Mean test error	$\#$ of variables selected		
		${\widehat{{\varvec{\beta }}}}_{1}$	${\widehat{{\varvec{\beta }}}}_{2}$	${\widehat{{\varvec{\beta }}}}_{3}$
CISESIR	0.005 (0.0218)	352.26	352.26	352.26
CISELDA	0.002 (0.0147)	297.63	297.63	297.63
MGSDA	0.007 (0.0024)	4.30	4.30	4.30
PLDA	0.058 (0.0094)	6774.9	5225.3	5476.8
MSDA	–	–		

Averages are over 50 repetitions of randomly splitting the data into training (99 observations) and testing (48 observations). Reported average error rates are obtained from the test sets

#### Riboflavin production data

Here, we apply the proposed method on data with continuous response variable. The data concerns riboflavin (vitamin B2) production with B. subtilis. We obtained the data from [37]. Please refer to the Data Availability Study for where to download the riboflavin data. There is a single real-valued response variable, which is the logarithm of the riboflavin production rate, and $p = 4088$ (co)variables that measure the logarithm of the expression level of 4088 genes; these gene expression profiles were normalized using the default in the R package affy [38]. The data consist of $n = 71$ samples that were hybridized repeatedly during a fed-batch fermentation process in which different engineered strains and strains grown under different fermentation conditions were analyzed. We refer interested readers to [37] for more details.

**Fig. 3:**
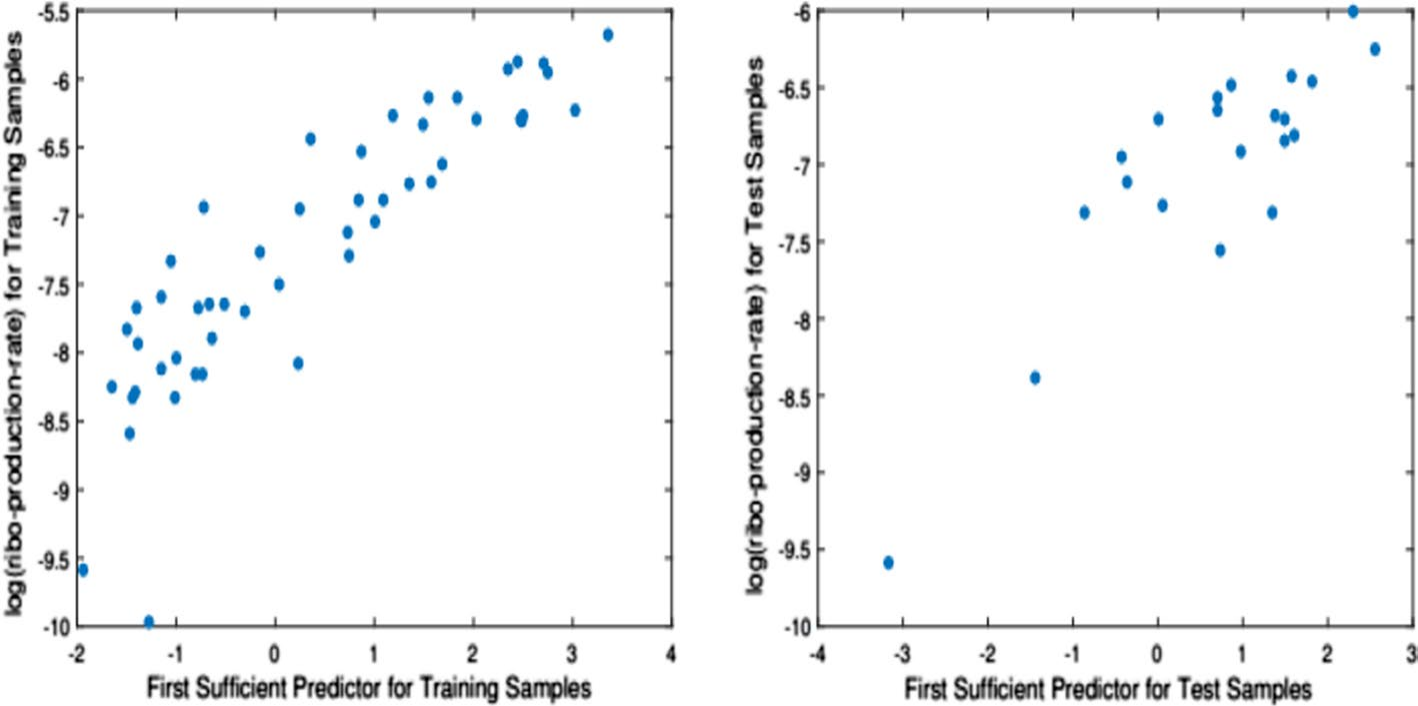
Riboflavin production data: left panel plot is the sufficient summary plot $(Y_{\text{train}}$ versus $\widehat{\mathbf{v }}_1^{\top }{{\mathbf{X}}}_{\text{train}})$ for the 50 training samples; right panel is the sufficient summary plot $(Y_{\text{test}}$ versus $\widehat{\mathbf{v }}_1^{\top }{{\mathbf{X}}}_{\text{test}})$ of the 21 test samples

Next, we estimate the structural dimension, which we find to be ${\widehat{d}} = 1$. Following, we randomly split the data into 50 training and 21 test samples. Then, we apply the proposed method on the training samples to obtain an estimate of the direction that span the central subspace, $\widehat{\mathbf{v }}_1$, and project both the training and the test samples to this estimated direction to obtain the corresponding sufficient predictors, $\mathbf{v }_1^{\top }{{\mathbf{X}}}_{\text{train}}$ and $\mathbf{v }_1^{\top }{{\mathbf{X}}}_{\text{train}}$. Figure 3 depicts the sufficient summary plots. We also repeated the foregoing procedure 50 times and counted the number of times each gene was selected, i.e. had a corresponding non-zero estimated coefficient in $\widehat{\mathbf{v }}_1$. We find that the following nine genes were selected in 80% of the replications: XHLA$\_$at, YCGO$\_$at, YHDX$\_$r$\_$at, YRZI$\_$r$\_$at, YTGD$\_$at, YCKE$\_$at, YXLD$\_$at, YCDH$\_$at, GAPB$\_$at.

## Summary and conclusion

We have introduced a novel sparse estimation method for the population reduction vectors in semi-parametric multi-index models using the sliced inverse regression [1]. Unlike most existing methods in this literature that follow the sequential estimation fashion, our proposed method yields simultaneous estimation of the reduction vectors. The estimated dimension reduction matrix is row-sparse and thus leads to coordinate-independent sparse estimates, in the sense that the selected predictors are the same under any orthogonal transformation of the reduction vectors that span the subspace of interest, making it appealing for variable screening. The proposed method extends the scope of the popular sliced inverse regression for dimension reduction [1] to the high-dimensional setting. We carried out extensive simulations and applications to assess the effectiveness of the proposed method. Relative to other state of the art methods in the literature, our numerical experiments show that our proposed method is competitive in prediction performance, and generally yield smaller false positive rates (FPRs) with respect to variable selection.

The proposed method was applied to three real datasets including data from a depression study aimed at identifying metabolites that differentiate patients with major depressive disorder (MDD) symptoms from patients without MDD symptoms. Our results show that a number of metabolites including some known to be associated with major depression are enriched in the set of metabolites selected by our method.

## Data Availability

We use publicly available data. The metabolomics data on major depressive disorder were obtained from the Metabolomics Workbench (http://www.metabolomicsworkbench.org/data,study number ST000063). The Ribflavin production data was obtained from https://www.annualreviews.org/doi/suppl/10.1146/annurev-statistics-022513-115545(riboflavin.csv).

## Abbreviations

AUC: Area under receiver operating characteristic curves
TPR: True positive rate
FPR: False positive rate

## References

[1] Li K-C (1991). Sliced inverse regression for dimension reduction. J Am Stat Assoc.

[2] Cook RD, Weisberg S (1991). Discussion of “sliced inverse regression for dimension reduction”. J Am Stat Assoc.

[3] Ni L, Cook RD, Tsai C-L (2005). A note on shrinkage sliced inverse regression. Biometrika.

[4] Li L (2007). Sparse sufficient dimension reduction. Biometrika.

[5] Bondell H, Li L (2009). Shrinkage inverse regression estimation for model-free variable selection. J R Stat Soc B.

[6] Chen X, Zou C, Cook RD (2010). Coordinate-independent sparse sufficient dimension reduction and variable selection. Ann Stat.

[7] Li L, Yin X (2008). Sliced inverse regression with regularizations. Biometrics.

[8] Cook RD (2004). Testing predictor contributions in sufficient dimension reduction. Ann Stat.

[9] Yu Z, Zhu L, Peng H, Zhu L (2013). Dimension reduction and predictor selection in semiparametric models. Biometrika.

[10] Wang T, Chen M, Zhao H, Zhu L (2018). Estimating a sparse reduction for general regression in high dimensions. Stat Comput.

[11] Lin Q, Zhao Z, Liu JS (2019). Sparse sliced inverse regression via lasso. J Am Stat Assoc.

[12] Tan KM, Wang Z, Zhang T, Liu H, Cook RD (2018). A convex formulation for high-dimensional sparse sliced inverse regression. Biometrika.

[13] Qian W, Ding S, Cook D (2019). Sparse minimum discrepancy approach to sufficient dimension reduction with simultaneous variable selection in ultrahigh dimension. J Am Stat Assoc.

[14] Cook RD (2007). Fisher lecture: dimension reduction in regression. Stat Sci.

[15] Cook RD, Forzani L (2008). Principal fitted components for dimension reduction in regression. Stat Sci.

[16] Yin X, Hilafu H (2015). Sequential sufficient dimension reduction for large p, small n problems. J R Stat Soc Ser B.

[17] Hilafu H, Yin X (2017). Sequential sufficient dimension reduction for large p, small n problems. J Comput Graph Stat.

[18] Yu Z, Dong Y, Shao J (2016). On marginal sliced inverse regression for ultrahigh dimensional model-free feature selections. Ann Stat.

[19] Lin Q, Zhao Z, Liu JS (2018). On consistency and sparsity for sliced inverse regression in high dimensions. Ann Stat.

[20] Li L, Wen XM, Yu Z (2020). A selective overview of sparse sufficient dimension reduction. Stat. Theory Relat. Fields.

[21] Kent J (1991). Discussion of Li (1991). J Am Stat Assoc.

[22] Cook RD, Yin X (2001). Dimension-reduction and visualization in discriminant analysis. Aust N Z J Stat.

[23] Cook RD, Ni L (2005). Sufficient dimension reduction via inverse regression: a minimum discrepancy approach. J Am Stat Assoc.

[24] Candes E, Tao T (2007). The dantzig selector: statistical estimation when *p* is much larger than *n*. Ann Stat.

[25] Cai T, Liu W, Luo X (2011). A constrained *l*_1_ minimization approach to sparse precision matrix estimation. J Am Stat Assoc.

[26] CVX-Research: Cvx: Matlab software for disciplined convex programming, version 2.0. http://cvxr.com/cvx 2012.

[27] Grant M, Boyd S. Graph implementations for nonsmooth convex programs. In: Blondel, V., Boyd, S., Kimura, H. (eds.) Recent advances in learning and control. Lecture Notes in Control and Information Sciences. Springer-Verlag Limited, pp. 95–110;2008.

[28] Gaynanova I, Booth JG, Wells MT (2016). Simultaneous sparse estimation of canonical vectors in the *p* > > *n* setting. J Am Stat Assoc.

[29] Mai Q, Yang Y, Zou H (2019). Multiclass sparse discriminant analysis. Stat Sin.

[30] Cai T, Liu W (2011). A direct estimation approach to sparse linear discriminant analysis. J Am Stat Assoc.

[31] Witten D, Tibshirani R (2011). Penalized classification using Fisher’s linear discriminant. J R Stat Soc B.

[32] Hasler G, van der Veen J, Tumonis T, Meyers N, Shen J, Drevets W (2007). Reduced prefrontal glutamate/glutamine and -aminobutyric acid levels in major depression determined using proton magnetic resonance spectroscopy. Arch Gen Psychiatry.

[33] Cotter DR, Pariante CM, Everall IP (2001). Glial cell abnormalities in major psychiatric disorders: the evidence and implications. Brain Res Bull.

[34] Rajkowska G, Miguel-Hidalgo JJ, Wei J, Dilley G, Pittman SD, Meltzer HY, Overholser JC, Roth BL, Stockmeier CA (1999). Morphometric evidence for neuronal and glial prefrontal cell pathology in major depression see accompanying editorial, in this issue. Biol Psychiat.

[35] Graveley BR, Brooks AN, Carlson J, Duff MO, Landolin JM, Yang L, Artieri CG, van Baren MJ, Boley N, Booth BW, Brown JB, Cherbas L, Davis CA, Dobin A, Li R, Lin W, Malone JH, Mattiuzzo NR, Miller D, Sturgill D, Tuch BB, Zaleski C, Zhang D, Blanchette M, Dudoit S, Eads B, Green RE, Hammonds A, Jiang L, Kapranov P, Langton L, Perrimon N, Sandler JE, Wan KH, Willingham A, Zhang Y, Zou Y, Andrews J, Bickel PJ, Brenner SE, Brent MR, Cherbas P, Gingeras TR, Hoskins RA, Kaufman TC, Oliver B, Celniker SE (2011). The developmental transcriptome of drosophila melanogaster. Nature.

[36] Frazee AC, Langmead B, Leek JT (2011). Recount: a multi-experiment resource of analysis-ready RNA-seq gene count datasets. BMC Bioinform.

[37] Buhlmann P, Kalisch M, Meier L (2014). High-dimensional statistics with a view toward applications in biology. Annu Rev Stat Appl.

[38] Gautier L, Cope L, Bolstad B, Irizarry R (2004). Affy analysis of affymetrix genechip data at the probe level. Bioinformatics.

